# Can behavioural change interventions improve self-efficacy and exercise adherence among people with Parkinson’s? A systematic review protocol

**DOI:** 10.12688/hrbopenres.13474.2

**Published:** 2022-08-22

**Authors:** Leanne Ahern, Prof Suzanne Timmons, Prof Sarah E Lamb, Dr Ruth McCullagh

**Affiliations:** 1 Department of Physiotherapy, School of Clinical Therapies, University College Cork, Cork, Ireland; 2 Centre for Gerontology and Rehabilitation, School of Medicine,, University College Cork, Cork, Ireland; 3 College of Medicine,, University of Exeter, Exeter, UK

**Keywords:** Parkinson’s, exercise self-efficacy, behavioural change interventions, quality of life, exercise adherence

## Abstract

**Background *:*** People with Parkinson’s (PwP) have a higher tendency to adopt sedentary lifestyle behaviours and have lower physical activity levels compared to their healthy peers. Previous research has indicated that personal factors including poor outcome expectation and low self-efficacy are stronger predictors of exercise adherence than disease severity. ***: *** The purpose of this review is to synthesize the best available evidence on interventions that encompass self-management strategies to overcome barriers to exercise and improve self-efficacy and exercise adherence among PwP.

**Methods *: *** The following databases will be searched using a comprehensive search strategy: EBSCO, Medline, CINAHL, Web of Science, PubMed, Embase, Scopus, Google Scholar and Cochrane Library from database inception to 2020. Interventional studies including behavioural change interventions will be included in this review. The title, abstract and full-text screening will be conducted by two independent reviewers. The Joanne Briggs Institute Checklist will be used to assess the quality of each included study. Data will be extracted by two independent reviewers. The outcomes of interest will be self-efficacy outcomes and measures of exercise adherence. A systematic narrative synthesis will be conducted using a framework analysis, applying the Theoretical Domains Framework and Behaviour Change Wheel, producing findings focusing on practice-orientated outcomes. Presentation of data will include tables and text summarizing the characteristics and findings of the eligible studies. Data synthesis and statistical analysis will be performed in Review manager 5.3. The quality of evidence will be reviewed using the GRADE criteria.

**Discussion *: *** The review will comprehensively synthesize the  available evidence on interventions to enhance self-efficacy, improve quality of life, physical function,  ultimately improving exercise adherence among PwP and provide invaluable information for healthcare professionals.. This review will make recommendations for appropriate self-management strategies for maximum effect and may have implications for policy and practice regarding enhancing self-efficacy and long-term exercise adherence among PwP.

## Introduction

Parkinson’s is the second most common neurological condition globally. This neurodegenerative condition effects the basal ganglia, leading to progressive movement disorders which with time become more disabling ^1^. Key motor features associated with Parkinson’s are tremor, rigidity (muscle stiffness), akinesia (difficulty initiating movement), bradykinesia (slow movements) and postural instability ^2^. There are also many non-motor features associated with Parkinson’s including apathy, depression, pain, fatigue, sleep disorders, cognitive impairment, and autonomic dysfunction ^3^. The combination of these motor and non-motor features can result in reduced mobility, reduction in quality of life and loss of function ^4^. As a result, people with Parkinson’s (PwP) have a higher tendence to adopt sedentary lifestyle behaviours and have lower levels of physical activity compared to their healthy peers ^4, 5^.

### Exercise, physical activity, and Parkinson’s

The advantages of regular physical activity are extensive with research suggesting that PwP benefit from physical activity in multiple ways including improvements in general health, disease-specific improvements and potentially disease-modifying effects ^6^

Physical activity is characterized as any bodily movement produced by skeletal muscles resulting in energy expenditure ^7^ including unstructured or incidental movement. The term exercise often interchangeably used with physical activity ^8^. However, exercise is a subcategory of physical activity ^7^. Exercise is physical activity that is planned, structured, repetitive and purposeful with the aim to improve or maintain one or more components of physical fitness (cardiorespiratory endurance, muscular strength, muscular endurance, flexibility and body composition) ^7^.

The role of exercise in the management of Parkinson’s is well-documented. The majority of exercise interventions for PwP focus of resistance training, balance, aerobic exercise, and flexibility conducted in an exercise or rehabilitative setting ^9^. A meta-analysis conducted by Choi *et al.* ^10^ investigated the effects of exercise therapies on PwP. Exercise therapies including walking ^11– 13^, strength and flexibility ^14– 17^, balance ^18, 19^, aerobic ^20– 23^ and combined exercise ^24– 28^ were shown to improve balance, walking speed, exercise tolerance, gait function, aerobic capacity, motor control, physical functioning, muscular strength and flexibility among PwP ^10^. However, exercise therapies did not show a significant effect on the non-motor symptoms. They concluded that exercise therapy is more effective for the motor symptoms rather than the non-motor symptoms of PwP ^10^.

However, Tennigkeit *et al.* ^29^ conducted a systematic review including 24 studies which discussed the benefits of exercise and self-management education for PwP from Sweden and Germany. Self-management education interventions included interactive group sessions, educations sessions for PwP and family members, educational video clips, role playing and handouts and self-monitoring techniques (using diaries for fluctuation in symptoms). They reported positive outcomes for health-related and general quality of life (QoL) ^30– 40^, depression ^30, 31, 33, 35– 39, 41, 42^, self-efficacy ^30, 35, 41, 43^, and functional mobility ^33, 43, 44^, suggesting the benefit of behavioural change interventions for improving the non-motor symptoms of PwP.

Despite the clear benefits of exercise and physical activity for PwP, only 30% achieve recommended activity levels, some are inactive for 70% of the day and most are less active than their age-matched peers ^45^. Recently, studies have shown that exercise may have protective effects associated with the basal ganglia (known as neurogenesis) which results in improvement in dopamine transmission, increased cerebral blood flow and new formation of neuronal synapses which in turn can improve motor function ^46^. Neurogenesis can result in a slowed progression of Parkinson’s and improvements in motor control, particularly when exercise is carried out at vigorous intensities ^46– 48^.

In addition to this, a study conducted by Sajatovic *et al.* ^41^ investigated the changes in depression in PwP (with depression) between a combined group exercise and self-management program and a self-directed individual exercise and self-management program. They reported no significant changes in apathy or anxiety in both groups. However, both groups displayed modest within group improvement in cognition, while the combined group showed additional significant improvements in depression ^41^. There was no significant differences between groups. This indicates that behavioural self-management strategies such as group education and peer support may improve non-motor features such as depression in PwP.

### Barriers to exercise in PwP

While good compliance can be achieved with prescribed exercise programmes with supervision within a clinical trial this does not completely translate to similar compliance during everyday life. Schootemeijer *et al.* ^49^ conducted a comprehensive review discussing the various barriers to exercise faced by PwP. They discussed barriers including non-motor factors (anxiety, depression, fatigue, and apathy), personal factors (low self-efficacy, fear of falling, low outcome expectation and lack of time) and environmental factors (lack of social support, lack of exercise partner, poor accessibility, bad weather, financial burden, cultural challenges, awareness of moving in a crowded environment, and discomfort of seeing advancing symptoms of peers) ^49^.

Although PwP experience increasing difficulties engaging in exercise as the disease progresses, previous research has indicated that personal factors including poor outcome expectation and low self-efficacy are stronger predictors of exercise adherence than disease severity ^50^.

In terms of exercise, self-efficacy is an individual’s confidence or belief that they can successfully engage in physical activity or exercise ^51, 52^. Exercise self-efficacy can be categorized into performance self-efficacy (beliefs about performing exercises) or beliefs in overcoming barriers ^53, 54^. Exercise self-efficacy determines the type of exercise an individual partakes in, their effort level, and their long-term exercise adherence when they face barriers to participation ^53, 55^ A meta – analysis conducted by Higgins *et al.* ^54^ reported that short-term exercise interventions (duration between two - eight weeks) were more effective for enhancing performance efficacy. In contrast interventions that included long-term strategies providing opportunities for individuals to experience and successfully conquer barriers over a longer period were more effective for enhancing confidence in overcoming barriers to exercise ^54^. This indicates the potential benefits of integrating long-term strategies into behavioural change interventions to promote long-term adherence to physical activity and exercise among PwP.

### Behavioural change

Adapting health behaviour in terms of changing from a sedentary lifestyle to a more physically active lifestyle is a complex process ^56^. Merely informing individuals about the benefits of physical activity has been shown as inadequate to maintaining behavioural change ^56, 57^. In order to assist behavioural change in PwP disease-specific counselling and coaching may be required ^58^. Behavioural change interventions are complex and involved many cooperating components ^59^. These psychology-focused interventions try to facilitate more constructive health behaviours ^60^. Particular strategies are utilized to promote behaviour change; some interventions are tailored to enhancing physical activity engagement by identifying barriers and problem solving ^61^. While others prompt individuals to track their sedentary behaviour as a method of changing behaviour ^61^. These interventions utilize theories of behaviour and behaviour change to inform particular therapeutic strategies ^62^.

Speelman *et al.* ^63^ studied the long-term effect of including behavioural change interventions (coaching, goal setting, use of activity monitors) into a multi-facet exercise program for PwP. They reported improvements in physical activity level for all subgroups of PwP ^63^. While Ellis *et al*. ^64^ investigated the effects of short daily interactions (five minutes/day) with a virtual exercise coach to encourage walking (monitored by a pedometer) among PwP. The interactions discussed progression of short- and long-term goals, collaborative problem solving to overcome barriers and positive support ^64^. They reported excellent retention rate in the walking program and improvements in gait after one month. However, due to the short duration of the intervention the long-term effects of adherence and occurrence of behaviour change are unknown ^64^.

### Theoretical Domains Framework (TDF)

The TDF is a combined theoretical framework which was created from 128 theoretical constructs and 33 behaviour theories ^65^. The TDF has been used in implementation research ^65^ to perform a process evaluation of randomized trials to further understand the effect of implementing evidence ^66^, as guidance on identifying behaviour change techniques ^62, 67^, to identify influences on behaviours ^68– 72^, and systematic intervention design ^73– 75^. 

The TDF consists of 14 domains subcategorized into personal factors, social factors and environmental factors ( Table 1). The TDF has previously been correlated with a simpler model of behaviour known as the COM-B ^67, 76^. The main principle of this model is that capability, opportunity, and motivation interact to produce behaviour. Whereas the TDF provides a more in-depth insight of psychological capability and reflective motivational processes ^65^.

**Table 1.** Theoretical Domains Framework domains and definitions ^65^.

TDF Domain	Definition
**Knowledge**	Awareness of the existence of something
**Skills**	Ability or proficiency acquired through practice
**Memory, attention, and** ** decision processes**	Ability to retain information, focus selectively on aspects of the environment and choose between two or more alternatives
**Behaviour regulation**	Anything aimed at managing or changing objectively observed or measured actions
**Beliefs about capabilities**	Acceptance of the truth, reality or validity about an ability, talent, or facility that a person can put to constructive use
**Beliefs about consequences**	Acceptance of the truth, reality, or validity about outcomes of a behaviour in a given situation
**Social/ professional role** ** and identity**	Behaviours and displayed personal qualities of an individual in a social or work setting
**Emotion**	A complex reaction pattern, involving experiential, behavioural, and physiological elements, by which the individual attempts to deal with a personally significant matter or event
**Intentions**	Conscious decision to perform a behaviour or a resolve to act in a certain way
**Reinforcement**	Increasing the probability of a response by arranging a dependent relationship, or contingency, between the response and a given stimulus
**Goals**	Mental representations of outcomes or end states that an individual wants to achieve
**Optimism**	Confidence that things will happen for the best or that desired goals will be attained
**Environmental context** ** resources**	Any circumstance of a person’s situation or environment that discourages or encourages the development of skills and abilities, independence, social competence, and adaptive behaviour
**Social Influences**	Interpersonal processes that can cause individuals to change their thoughts, feelings, or behaviours

In order to motivate individuals with Parkinson’s to remain physically active outside a clinical setting it is important to identify self-management strategies to overcome these barriers, improve self-efficacy and promote physical activity among PwP. To the best of our knowledge this is the only review exploring the effectiveness of behaviour change interventions on self-efficacy and long-term exercise adherence among PwP. The findings of this review will make recommendations for appropriate self-management strategies and may have implications for policy and practice.

## Review objectives

The purpose of this review is to comprehensively synthesize the best available evidence on behaviour change interventions that encompass self-management strategies to over barriers to exercise and improve exercise adherence among PwP.

Specifically, the objectives are to:

- Examine self-management strategies to overcome barriers to exercise among PwP.
- Determine the effectiveness of behavioural change interventions aimed to improve exercise self-efficacy, QoL and physical function and exercise adherence among PwP.
- Identify strategies to promote long-term exercise adherence among PwP.

## Research question

Specially, this review is aimed to answer the following questions:

1. Do behavioural change interventions improve exercise self-efficacy among PwP?

2. Do behavioural change interventions improve QoL and/or physical function among PwP?

3. Do behavioural change interventions improve exercise adherence/increase levels of physical activity among PwP?

## Methods

This protocol was designed in line with the methodological framework provided by the Joanna Briggs Institute (JBI) Reviewer’s Manual ^77^ and the Preferred Reporting Items for Systematic reviews and Meta-Analyses (PRISMA) guidelines ^78^. This review is registered with PROSPERO (ID: CRD42021293057). *Extended Data:* PRIMSA-P Checklist

### Inclusion and exclusion criteria

Studies to be included in this review must satisfy the inclusion criteria outlined in Table 2.

**Table 2:** Inclusion and exclusion criteria.

*Study* * Characteristics*	*Inclusion Criteria*	*Exclusion Criteria*
(i) Population, or participants and conditions of interest	• Community dwelling independently mobile people with Parkinson’s. • No limitations will be placed on the length of time since diagnosis or age. • Studies including people with Parkinson’s diagnosed with other comorbidities (e.g anxiety, depression, and diabetes) can be included. However, outcomes must focus on exercise self-efficacy and/or exercise uptake/adherence and not changes in the comorbidity. • Population will not be restricted to Ireland or the UK, articles from all countries will be examined.	If recruited participants: a) Do not have a diagnosis of Parkinson’s, or have a diagnosis of Atypical Parkinson’s b) Are current inpatients or had a recent hospital admission within the last 3 months. c) Are immobile or wheelchair-users, c) Involve severe visual or auditory impairment, serious medical conditions in major organs (heart, lung, or kidney) or other illnesses which prevent independent ambulation. d) Involve people with Parkinson’s who are identified as a high falls risk (fallers)
(ii) Intervention	• Any form of behavioural change intervention (e.g education, behavioural technology, or support groups) or support strategy to improve QoL, exercise self-efficacy or exercise uptake. For the purpose of this review *behavioural change intervention* will be defined as any psychology-focused intervention (used in conjunction with exercise or alone) ^60^. While *exercise self-* *efficacy* is defined as an individual’s confidence or belief that they can successfully engage in physical activity or exercise ^51, 52^	• The intervention does not include self- efficacy strategies or behavioural change interventions. • The intervention focuses solely on falls prevention
(iii) Comparator	• Comparator groups must include people with a Parkinson’s diagnosis • A specific intervention type will not be defined for the purpose of inclusive. • Comparator groups including but not limited to exercise alone, usual care or waiting list will be included	• Comparator group including non- Parkinson’s individuals.
(iv) Outcomes of interest	• Outcomes reported at every time-points will be considered for this review. • Primary outcomes are self-efficacy measures (e.g Self-efficacy for exercise scale, Physical Activity Assessment Inventory), QoL (e.g PDQ-39. PDQ-8), physical function (e.g 6MWT, gait velocity), and measures of exercise adherence (e.g self-log, activity monitors).	Outcomes reported are not related to exercise adherence/uptake (i.e medication adherence, changes in anxiety and depression)
(v) Setting	Studies conducting interventions in the following settings will be included; community gyms/halls, community outpatient facilities, acute hospitals (if the intervention is conducted with community dwelling people with Parkinson’s), home environment or in any geographical setting globally.	Acute hospitals (if intervention is conducted with inpatients), Long-term care facilities.
(vi) Study design	Interventional studies: RCTs, quasi-experimental trials, pilot interventional studies, pre- and post- interventional studies, and feasibility studies.	Qualitative studies, observational studies, or systematic reviews
		Other: • Full-text articles are not available. • Papers are not published in English
(vii) Phenomenon of interest	The review will include studies that explore behavioural change strategies to enhance exercise self-efficacy, improve QoL, physical function and ultimately improve adherence to exercise among community dwelling individuals with Parkinson’s, including but not limited to behavioural interventions (motivational interviewing, goal setting and cognitive re-framing) and support strategies (peer and family education and support sessions).	

***Search strategy.*** Two independent reviewers (LA and RMcC) will conduct a search using the following electronic databases: *EBSCO, Medline, Cinhal, Web of science, PubMed, Embase, Scopus, Google Scholar, Cochrane Library.* Databases will be searched from inception to 2020. The search strategy was developed by the primary author (LA) and supported by a librarian with systematic review experience (VC). Two independent reviewers (LA and RMcC) will search the databases using the search terms showed in Table 3. Reference lists of related articles and relevant reviews will be checked to identify further eligible studies.

**Table 3.** Search strategy.

Databases: - EBSCO (Academic search complete and Psychinfo) - Medline - Cinahl - Web of Science - PubMed - Embase - Scopus - Google Scholar - Cochrane Library
Search keywords:
*1*. [“behavioural change intervention*” OR “behavioral change intervention*” OR “behaviour change technique*” OR “behavior change technique*” OR “cognitive behavioural therapy” OR “cognitive behavioral therapy” OR psychology OR “psychological therapy” OR “health behaviour*” OR “health behavior*”]
*2*. [self-efficacy OR “self efficacy” OR *“*physical activity self-efficacy *”* OR *“*physical activity self efficacy *”* OR *“*exercise self- efficacy *”* OR *“*exercise self efficacy” OR self-management]
*3*. 1 AND 2
*4*. [ *“*physical activit *” OR recreation OR sport OR exercise OR training OR fitness OR *“*physical therap* *”* OR rehabilitation]
*5*. 3 AND 4
*6*. [ *“*Parkinson’s Disease *”* OR “Parkinsons Disease” OR “Parkinson Disease” OR Parkinson’s OR Parkinson]
*7*. 5 AND 6

***Study records.*** Articles identified from the literature search will be uploaded to Endnote X8, a citation manager. Duplicates will be removed using the “remove duplicates” function, and manual screening of the results will be conducted to ensure accuracy (LA). Titles and abstracts of the identified articles will then be exported to Rayyan (LA), an electronic software designed to support article screening and allows collaboration between reviewers during the study selection process.

***Study selection.*** Two independent reviewers (LA and RMcC) will be involved in the study selection process through each phase of the review. Following the removal of duplicates, LA and RMcC will independently screen all titles and abstracts of the articles identified by the literature search. Studies not meeting the inclusion criteria will be excluded. Prior to the formal screening process, test screening questions will be developed based on the inclusion/exclusion criteria.

Subsequently, LA and RMcC will independently screen the full text articles identified from the previous stage to select the suitable studies. Reference lists of the included articles and previously conducted reviews in the area will be checked to identify any additional studies. Both LA and RMcC will independently screen any additional articles to determine their suitability. Any disagreement regarding inclusion will be resolved by a third reviewer (ST). A Preferred Reporting Items for Systematic Review and Meta-Analysis (PRISMA) flow diagram will display the study selection process and summarise the inclusion and exclusion of studies at each stage of the review by providing reason for exclusion.

### Data collection and extraction

Two independent reviewers (LA and RMcC) will extract data from each eligible study and conduct the risk of bias assessment. Reviewers will perform practice extraction exercises prior to the formal extraction to ensure consistency. Any disagreement regarding extraction will be resolved by a third reviewer (ST) and a consensus achieved. If required, primary authors of studies will be contacted to provide further details. A data extraction template will be designed a priori. Data including study design, sample characteristics (size, gender, mean age) specific details about the intervention (type, duration and follow-up) and implementation methods, pre- and post-intervention outcome results, and theorical framework used (if applicable) will be extracted.

### Methodological quality of studies

To assess the potential risk of bias The Joanne Briggs Institute Checklist ^77^ for the corresponding study designs will be used for each eligible study.

Two independent reviewers (LA and RMcC) will assess the potential risk of bias of each article. Any disagreements will be resolved by a third reviewer (ST). In the incidence where data is missing, or information is not clear the primary authors will be contacted for clarification. Following the assessment, studies will be classified as a high, medium, low, or unclear risk of bias.

### Assessing the quality of evidence

The quality of evidence will be assessed using the Grades of Recommendation, Assessment, Development and Evaluation (GRADE) approach ^79^. This involves assessing the quality of evidence using a specific points system to upgrade or downgrade the ratings for each quality characteristic. Evidence can be downgraded one level for serious limitations or two levels for very serious limitations depending on the assessment for five characteristics: limitation in study design and conduct, inconsistent results across studies, indirectness of evidence with respect to study design, populations, interventions, comparisons or outcomes, imprecision of the estimates of the effect and publication bias. Evidence can be upgraded depending on the assessment of the following three characteristics; large magnitude of effect, plausible confounding that would reduce the effect, and dose-response gradient ^80– 83^.

Two independent reviewers will assess the quality of each eligible articles (LA and RMcC). Any disagreement will be resolved by a third reviewer (ST) and a consensus achieved. In the incidence where information is not clear the primary authors will be contacted for clarification.

### Narrative synthesis and analysis of the data 

Initially, a narrative analysis will be completed . If data is missing or incomplete, we will contact the author by email for the information. Data will be presented using tables and text summarizing the characteristics and findings. A narrative synthesis of the findings will be completed to investigate the association and findings between the included studies. Patterns demonstrating the effectiveness of the interventions will be discussed, including the participants, the intervention, and its impact on the outcomes. Outcomes will include subjective measures of impact including self-efficacy and quality of life, as well as objective measures of physical / functional gains (e.g 6MWT, gait velocity), and measures of exercise adherence (e.g self-log, activity monitors).

The interventions will be mapped to the Theoretical Domains Framework (TDF) and Behaviour Change Wheel (BCW), producing findings focusing on practice-orientated outcomes. The TDF includes fourteen domains related to the psychology of behaviour change ^84^. While the BCW focuses on the success of implementing interventions by coordinating change interventions with behavioural barriers; a person’s opportunity, capability and motivation interconnects and influence their behaviour (COM-B) ^85^. The effectiveness of the mapped interventions will be evaluated further in the context of the models.

The data will be assessed for eligibility for meta-analysis, exploring heterogeneity of populations, interventions, and outcome.

***Meta-analytical approach***. Statistical analysis will be performed by using the Cochrane Review Manager (RevMan 5.3) software. The mean difference and the 95% confidence interval (CI) will be used as an effective size for the combined analysis, with p ≤ 0.05 considered as statistically significant. 

**Assessment of heterogeneity**

If significant methodological, statistical, and clinical heterogeneity is detected, results will not be reported as the pooled effect estimate in a meta-analysis. Heterogeneity will be identified by visual inspection of the forest plots and by conducting a χ ^2^ test with a significance level of α = 0.1. Heterogeneity will also be examined using the I ^2^ statistic as recommended by Higgins ^86, 87^. The I ^2^ statistic will quantify inconsistency across the included studies in order to assess the impact of heterogeneity on the meta-analysis. An I ^2^ statistic ≥ 75% signifies a substantial level of inconsistency ^88^. If heterogeneity is determined, individual studies and subgroup characteristics will be examined to identify potential reasons. 

Data will be summarized by means of a random-effects model with due consideration of the whole distribution of effects by presenting a prediction interval. Analyses will be conducted according to the guidelines outlined in the Cochrane Handbook for Systematic Reviews of Interventions ^88^. 

**Subgroup analysis**

Subgroup analyses will be completed to determine the effectiveness of behavioural change strategies on quality of life based on the type of strategy.

**Assessment of reporting biases**

If ten or more of the included studies investigate the same outcome, funnel plots will be used to assess small study effects and evaluate potential publication bias using the Egger test in Stata 13.

**Sensitivity analysis**

A sensitivity analyses will be performed to explore the influence of the following factors (if applicable) on effect sizes.

- Restricting analysis to high-powered, larger studies
- Restricting analysis to low risk of bias studies
- Restricting analysis to studies including a control group.

To test the robustness of the results analysis will be repeated using different statistical models (random-effects and fixed-effect models) and different measures of effect size (odds ratio and relative risk).

## Dissemination of results

The systematic review will be disseminated in a peer-reviewed journal and the results will be presented both locally to physiotherapy clinical colleagues, and at international conferences. The dataset created during the study will be available from the corresponding author upon request.

## Amendments

Any amendments to this protocol will be described in a table including the date of each amendment as well as a description of and rationale, this will be documented in a note to a later publication (section “Differences between protocol and review”). The PROSPERO register will remain updated with the protocol and any amendments made.

## Ethics approval and consent to participate

Ethical approval is not required for this study as it will not involve conducting experimental research or include identifying personal data.

## Study status

The systematic review protocol was finalised in November 2021 and the database search was conducted in December 2021. Full-text screening will be completed in January 2022. It is anticipated the review will be completed in September 2022.

## Discussion

Self-efficacy and attitudes towards exercise are linked in a linear relationship ^55, 89^. Exercise self-efficacy increases with mastery experiences, as individual become more experienced with exercise. However, self-efficacy also plays an important role in maintaining motivation to exercise ^52^. While the body of evidence supporting behavioural change interventions displays a positive effect of self-efficacy there is a need to pool evidence from trials to accurately determine the treatment effect of these different interventions.

This will be the first review of behavioural change interventions implemented to enhance self-efficacy and improve exercise adherence among PwP. By exploring this, the findings of this review will provide invaluable information for healthcare professionals. Additionally, this review will make recommendations for appropriate self-management strategies for maximum effect and may have implications for future policy and practice regarding enhancing self-efficacy and long-term exercise adherence among PwP.

## Data availability

No data are associated with this article.
