# Supplementary material for: Can behavioural change interventions improve self-efficacy and exercise adherence among people with Parkinson’s? A systematic review protocol
Source: HRB Open Res. 2022 Aug 22;5:15. Originally published 2022 Feb 23. [Version 2] doi: 10.12688/hrbopenres.13474.2 (PMC9440372; doi:10.12688/hrbopenres.13474.2)
Supplement: Peer review history

# Peer review: Reviewer response for version 2

**DOI:** 10.21956/hrbopenres.14855.r32720

**Published:** 2022-09-02

**Authors:** Katherine Baker

**Affiliations:** 1 Department of Sport, Exercise and Rehabilitation, Northumbria University, Newcastle upon Tyne, UK

Thank you for sharing the revisions to your protocol. The additional detail and clarification have strengthened the article which is an important addition to the literature.

Is the study design appropriate for the research question?

Yes

Is the rationale for, and objectives of, the study clearly described?

Yes

Are sufficient details of the methods provided to allow replication by others?

Partly

Are the datasets clearly presented in a useable and accessible format?

Not applicable

Reviewer Expertise:

Physiotherapy, Parkinson's, physical activity and exercise

I confirm that I have read this submission and believe that I have an appropriate level of expertise to confirm that it is of an acceptable scientific standard.

# Peer review: Reviewer response for version 1

**DOI:** 10.21956/hrbopenres.14686.r31803

**Published:** 2022-04-25

**Authors:** Ailish Malone

**Affiliations:** 1 RCSI University of Medicine and Health Sciences, Dublin, Ireland

Thank you for the invitation to review this systematic review protocol. The protocol addresses an important gap in current evidence synthesis and proposes robust methods to achieve its aims. 

 The Introduction clearly conveys the state of the evidence in favour of the myriad benefits of exercise in Parkinson’s, and the problem of inactivity. The review refers almost exclusively to “exercise” but the interventions might have an effect on habitual physical activity, too. Does the review intend to focus exclusively on *exercise* (structured, planned, repetitive, intentional), as distinct from *physical activity* (also including unstructured / incidental movement)? (I see that “physical activity” is a search term but the rest of the review refers to “exercise”.) 

 The last paragraph under the subheading “Exercise in Parkinson’s” presents the Sajatovic study, which compared a **group**-based exercise and self-management program with an **individual** exercise and self-management program. Participants in the **group** program showed additional improvements in depression scores, but were these differences seen within-group or between-group? Is the key difference, then, the mode of delivery (group v individual) rather than the components of the intervention and if so, what bearing might this have on the review?

 Section “Barriers to exercise in PwP” – this section clearly communicates the problems with the translation of evidence for exercise into the real-world setting. The last paragraph could include a summary sentence. What are the implications for the review from the outcomes of short-term and long-term strategies?

**Methods**

 The methods are mostly clear and follow the expected process for a systematic review. Some clarifications:

- Inclusion and exclusion criteria
  For (i) Population, exclusion criterion b: please amend “wheelchair-bound” to “wheelchair user” (“wheelchair user” is the preferred, more inclusive term)
  (iv) Setting: The study excludes acute hospitals. I see the rationale for this for current hospital inpatients or people who had a recent admission. However, would this exclusion apply if an outpatient intervention for community-dwelling people with Parkinson’s happened to be delivered at an acute hospital? The service arrangements for delivery of outpatient care to people with Parkinson’s might vary among health services and interventions meeting criteria (ii) could occur at an acute, specialist neurological hospital. If a study’s population, intervention, and outcome(s) meet the inclusion criteria, should it not be included, irrespective of where it took place?
- Data extraction – do the authors have a plan for identifying and handling duplicate data (for example, where the same trial produced multiple papers with secondary analyses?)

The final article needs a thorough proofread as several minor errors remain in this version.

Is the study design appropriate for the research question?

Yes

Is the rationale for, and objectives of, the study clearly described?

Yes

Are sufficient details of the methods provided to allow replication by others?

Yes

Are the datasets clearly presented in a useable and accessible format?

Not applicable

Reviewer Expertise:

NA

I confirm that I have read this submission and believe that I have an appropriate level of expertise to confirm that it is of an acceptable scientific standard.

# Response

**Published:** 2022-07-16

**Authors:** Leanne Ahern

**Affiliations:** University College Cork, Ireland

Thank you so much for taking the time to review our manuscript, and for your constructive comments. Responses and subsequent changes to the manuscript are detailed below.

**Comment: **The Introduction clearly conveys the state of the evidence in favour of the myriad benefits of exercise in Parkinson’s and the problem of inactivity. The review refers almost exclusively to “exercise” but the interventions might have an effect on habitual physical activity, too. Does the review intend to focus exclusively on exercise (structured, planned, repetitive, intentional), as distinct from physical activity (also including unstructured/incidental movement)? (I see that “physical activity” is a search term but the rest of the review refers to “exercise”.)

**Response: **Thank you for this comment, with was a factor in which we overlooked

**Action: **Paragraph added to the introduction regarding physical activity. This review intends to focus on both exercise and physical activity

**Comment: **The last paragraph under the subheading “Exercise in Parkinson’s” presents the Sajatovic study, which compared a group-based exercise and self-management program with an individual exercise and self-management program. Participants in the group program showed additional improvements in depression scores, but were these differences seen within-group or between-group? Is the key difference, then, the mode of delivery (group v individual) rather than the components of the intervention and if so, what bearing might this have on the review?

**Response: **Thank you for this comment as it is important that the information is clear for the readers. Upon further review of the paper, the differences that were reported in this review were within-group, therefore, highlighting that the differences are not solely related to mode of delivery.

**Action: **Rephrasing and more information provided to ensure clear message is conveyed regarding the results of this study

**Comment: **Section “Barriers to exercise in PwP” – this section clearly communicates the problems with the translation of evidence for exercise into the real-world setting. The last paragraph could include a summary sentence. What are the implications for the review from the outcomes of short-term and long-term strategies?

**Response: **Thank you for this comment

**Action: **Included summary sentence at end of paragraph

**Comment: **The methods are mostly clear and follow the expected process for a systematic review. Some clarifications:

 Inclusion and exclusion criteria

 For (i) Population, exclusion criterion b: please amend “wheelchair-bound” to “wheelchair user” (“wheelchair user” is the preferred, more inclusive term)

 (iv) Setting: The study excludes acute hospitals. I see the rationale for this for current hospital inpatients or people who had a recent admission. However, would this exclusion apply if an outpatient intervention for community-dwelling people with Parkinson’s happened to be delivered at an acute hospital? The service arrangements for delivery of outpatient care to people with Parkinson’s might vary among health services and interventions meeting criteria (ii) could occur at an acute, specialist neurological hospital. If a study’s population, intervention, and outcome(s) meet the inclusion criteria, should it not be included, irrespective of where it took place?

**Response: **Thank you for this comment, we agree that clarification is needed.

**Action: **The suggestions have been considered and amendments have been made regarding the inclusion criteria for the population and the setting.

# Peer review: Reviewer response for version 1

**DOI:** 10.21956/hrbopenres.14686.r31537

**Published:** 2022-03-11

**Authors:** Katherine Baker

**Affiliations:** 1 Department of Sport, Exercise and Rehabilitation, Northumbria University, Newcastle upon Tyne, UK

Thank you for the opportunity to review this manuscript. The proposed review will make an important contribution to the evidence base. 

 Abstract: Provides a useful summary and rationale for the review. Methods could include study designs that will be included. 

 Introduction: includes relevant and contemporary work in the area. This section clearly outlines the need to better understand ways of influencing behaviour change. As the Theoretical Domains Framework and Behaviour Change Wheel are proposed as the theoretical frameworks for the review, it would be appropriate to acknowledge them here. 

 Review objectives: exercise adherence is not identified as an outcome in the second objective, consider including this given the third objective which talks about the strategies but not adherence as an outcome. 

 Methodology: it is interesting that you propose a qualitative synthesis when the review inclusion criteria allows only quantitative study designs. Further explanation is needed on how this will be done. 

 There are typographical mistakes throughout, please review carefully.

Is the study design appropriate for the research question?

Yes

Is the rationale for, and objectives of, the study clearly described?

Yes

Are sufficient details of the methods provided to allow replication by others?

Partly

Are the datasets clearly presented in a useable and accessible format?

Not applicable

Reviewer Expertise:

Physiotherapy, Parkinson's, physical activity and exercise

I confirm that I have read this submission and believe that I have an appropriate level of expertise to confirm that it is of an acceptable scientific standard, however I have significant reservations, as outlined above.

# Response

**Published:** 2022-07-16

**Authors:** Leanne Ahern

**Affiliations:** University College Cork, Ireland

Thank you so much for taking the time to review our manuscript, and for your constructive comments. Responses and subsequent changes to the manuscript are detailed below.

**Comment:** Abstract: Provides a useful summary and rationale for the review. Methods could include study designs that will be included.

**Response: **We agree with this comment and believe it would provide important information to the readers

**Action: **Sentence “Interventional studies including behavioural change interventions will be included in this review” was included in the abstract

**Comment: **Introduction: includes relevant and contemporary work in the area. This section clearly outlines the need to better understand ways of influencing behaviour change. As the Theoretical Domains Framework and Behaviour Change Wheel are proposed as the theoretical frameworks for the review, it would be appropriate to acknowledge them here.

**Response: **We agree with this comment and admit it was an element which we overlooked. Thank you for this comment.

**Action: **Paragraphs regarding the Theoretical Domains Framework and Behaviour Change Wheel have been included in the introduction

**Comment: **Review objectives: exercise adherence is not identified as an outcome in the second objective, consider including this in the third objective which talks about the strategies but not adherence as an outcome.

**Response: **We agree with this comment as exercise adherence is referred to many times in the article.

**Action: **Exercise adherence included as an outcome in the second objective

**Comment: **Methodology: it is interesting that you propose a qualitative synthesis when the review inclusion criteria allows only quantitative study designs. Further explanation is needed on how this will be done.

**Response: **Thank you for this comment, we were not aware of the lack of transparency with regards to this.

**Action: **Methods section has been amended to a narrative synthesis with the addition of assessment for eligibility for meta-analysis
